# The globular heads of the C1q receptor regulate apoptosis in human cervical squamous carcinoma cells via a p53-dependent pathway

**DOI:** 10.1186/1479-5876-10-255

**Published:** 2012-12-26

**Authors:** Zheng-lin Chen, Ping-qing Gu, Kangsheng Liu, Ya-juan Su, Ling-juan Gao

**Affiliations:** 1 Clinical Laboratory, Jiangsu Provicial Official Hospital, Nanjing, 210024, China; 2 Department of Clinical Laboratory, State key Laboratory of Reproductive Medicine, Nanjing Maternity and Child Health Care Hospital Affiliated to Nanjing Medical University, Nanjing, 210004, China; 3 Clinical Laboratory, the Third Affiliated Hospital of Harbin Medical University, Harbin, 150080, China

**Keywords:** Globular heads of the human C1q receptor (gC1qR), Apoptosis, Mitochondria, p53, Human cervical squamous carcinoma cells

## Abstract

**Background:**

The globular heads of the human C1q receptor (gC1qR) localize predominantly to the mitochondrial matrix. gC1qR mediates many biological responses, including growth perturbation, morphological abnormalities and the initiation of apoptosis. The purpose of this study was to investigate the relationship between mitochondrial dysfunction, p53 status and gC1qR expression and the regulation of apoptosis in human cervical squamous carcinoma cells (C33a and SiHa).

**Methods:**

Here, gC1qR expression was examined in human cervical tissues using real-time PCR and Western blot analysis. Apoptotic death of C33a and SiHa cells was assessed by flow cytometric analysis that detected the subG1 population. Mitochondrial function was assessed via ROS generation, the content of cytosolic Ca^2+^, and the change in mitochondrial membrane potential (Δψm). The viability and migration of C33a and SiHa cells were detected via the water-soluble tetrazolium salt (WST-1) assay and the transwell assay, respectively.

**Results:**

gC1qR expression was decreased in cervical squamous cell carcinoma tissues compared with normal tissues. C33a and SiHa cells transfected with a vector encoding gC1qR displayed mitochondrial dysfunction and apoptosis, which was abrogated by the addition of a mutant form of p53 or p53 small interference RNA (siRNA). Furthermore, upon overexpression of gC1qR, cell viability and migration were significantly enhanced, and the apoptosis of C33a and SiHa cells were decreased when cells were treated with mutant p53 or p53 siRNA.

**Conclusions:**

These data support a mechanism whereby gC1qR induces apoptosis through the mitochondrial and p53-dependent pathways in cervical squamous cell carcinoma.

## Background

Cervical cancer is the most common malignant gynaecological disorder worldwide [1]. The lack of preventive strategies, early diagnostic methods, and effective therapies to treat recurrent cervical tumours creates a pressing need to understand its pathogenesis and to identify molecular markers and targets for diagnosis and therapy [2,3]. However, this involves the expression of both pro- and anti-apoptotic proteins that remain largely unknown.

The receptor for the globular heads of C1q, gC1qR/p33, is a ubiquitous and highly anionic cellular protein of 33 kDa that was initially identified and characterised as a receptor for the globular heads of C1q [4]. There is evidence that gC1qR mediates many biological responses, including inflammation, infection and immune regulation [5]. gC1qR-induced T-cell dysfunction involves the induction of suppressor of cytokine signaling (SOCS), a powerful inhibitor of cytokine signalling, which represents a novel mechanism of action [6]. Indeed, examples of such responses include growth perturbation, morphological abnormalities and initiation of apoptosis [7]. The present study assessed the effect of gC1qR on the apoptosis of cervical squamous carcinoma cells, and investigated the gC1qR-induced activation of the p53-dependent pathway.

## Materials and methods

### Reagents

The C33a and SiHa cervical squamous carcinoma cell lines were obtained from Boster Technology (Wuhan, HuBei, China). The human cervical epithelial cell line CRL2614 was purchased from American Type Culture Collection (ATCC). The phototope-HRP Western Blot Detection System was purchased from Cell Signaling Technology (Beverly, MA, USA). 2', 7'-dichlorodihydrofluorescein diacetate (H_2_DCFDA) was obtained from Molecular Probes. A Flow Cytometry Assay Kit was purchased from Invitrogen. Antibodies directed against gC1qR, p53 and actin were purchased from Santa Cruz (Santa Cruz, CA, USA) and Cell Signaling Technology. The plasmids pCB6+ p53 and pCB6+ p53 173 L encode wild-type (wt) and mutant p53 (mt), respectively, and were kindly supplied by Hangzhou Hibio Bio-tech Co., Ltd. The initial amino acid before mutation for pCB6 + p53 173 L were kqsqhmtev (164-172). p53 small interference RNA (siRNA) and negative siRNA (An unrelated gene siRNA as a negative control) were synthesized by Wuhan Genesil Biotechnology Co., Ltd (Wuhan, China). Cell culture supplies were purchased from Life Technologies (Gaithersburg, MD, USA).

### Tissue procurement and preparation

Human cervical cancer tissues were collected from 30 patients who underwent radical hysterectomy due to cervical carcinoma at Nanjing Maternity and Child Health Care Hospital between October 2007 and January 2010. Tumour specimens were obtained immediately after surgery. Local ethical approval was obtained before beginning this study and, where appropriate, tissues were collected with informed consent. The tumours and surrounding non-neoplastic tissues were reviewed by a pathologist and histologically confirmed.

### Cell culture and DNA transfection

Cells were cultured in Dulbecco’s modified Eagle’s medium/Ham’s F-12 medium containing 10% foetal bovine serum and 5 μg/mL insulin in a 37°C incubator with 5% CO_2_. Complementary DNA (cDNA) encoding gC1qR was cloned in frame using the BamHI/EcoRI sites into the pcDNA 3.1 expression plasmid (Invitrogen, Carlsbad, CA). The resulting gC1qR vector was then transfected into C33a and SiHa cells. 24 h after plating, cells were serum-starved for a further 24 h to synchronize cultures into quiescence. Following serum starvation, cells were transfected using LipofectAMINE™ reagent (Life Technologies, Inc.) according to the vendor’s protocol. Briefly, 0.05–1.5 μg/ml of plasmid DNA and 12 μg/ml LipofectAMINE™ were diluted in serum-free DMEM. After incubation for 30 min at 37°C, the DNA-liposome complexes were then added dropwise to each culture dish and incubated at 37°C, 5% CO_2_ for 12 h. Following transfection, cells were maintained in culture in serum-free DMEM. Reporter gene levels were normalised to total protein, and all results represent the average of triplicate experiments.

### Real-time quantitative polymerase chain reaction (Real-time PCR)

Total RNA was isolated from tissues by using Trizol reagent (Invitrogen) according to the manufacturer’s instructions. The isolated RNA was then DNase-treated and reverse-transcribed according to the manufacturer’s instructions. Real-time PCR was performed using ABI PRISM 7300 sequence detection system (Applied Biosystems) with the following thermal cycling conditions: 2 min at 50°C, 10 min at 95°C followed by a total of 40 cycles of 15 s at 95°C and 1 min at 60°C. All reactions were conducted in a 50 μL reaction volume in triplicate. β-actin was used as an internal control in all PCR experiments. Relative amounts of gC1qR mRNA were normalised to β-actin mRNA: 2^-ΔΔCT^=2^-(CT.gC1qR- CT.actin)Time x + (CT.gC1qR- CT.actin)Time 0^.

### Western blot analysis

After various treatments, cells were collected and placed in sample buffer, and then the cells were incubated in lysis buffer and protease inhibitor mixtures for 30 min on ice. The supernatants were collected by centrifugation at 13,000 × g at 4°C for 15 min. An equal amount of protein was separated by SDS-PAGE on a 10-15% polyacrylamide gel and then transferred onto a PVDF membrane. The transferred membranes were blocked for 1 h in 5% nonfat milk in PBST (PBS containing 0.05% Tween-20) and incubated with appropriate primary antibodies and horseradish peroxidase-conjugated secondary antibodies. The protein bands were visualised using the enhanced chemiluminescence (ECL) Western Detection System.

### Assay of intracellular ROS in C33a and SiHa Cells

C33a and SiHa cells were incubated with H_2_DCFDA (10 μM) under various conditions for 10 min in the dark and lysed with RIPA buffer in ice-cold conditions [8]. ROS levels were detected by fluorescence microscopy at an excitation wavelength of 488 nm and emission at 530 nm. A spectrofluorometer with a slit width of 5 nm was used to quantify the fluorescence in the supernatant. The results are shown as an increase in fluorescence intensity with respect to the normoxic untreated control after subtracting basal fluorescence.

### Measurement of intracellular Ca^2+^ concentration ([Ca^2+^]i)

Fluo-4 AM fluorescence was used to quantify the intracellular Ca^2+^ levels. The cells were resuspended in 1 mL of PBS and incubated with 5 mL of Fluo-4 AM 1 mM for 1 h. The fluorescence intensity was detected by a Beckman Coulter Paradigm™ Detection Platform at an excitation wavelength of 485 nm and an emission wavelength at 530 nm. Fluorometric measurements were performed in ten different sets and are expressed as the fold increase in fluorescence per microgram of protein compared with the control group.

### Measurement of mitochondrial membrane potential (Δψm)

JC-1 (Molecular Probes, Eugene, OR, USA) is a cationic mitochondria-specific fluorescent dye that was used to detect the loss of mitochondrial membrane potential (Δψm) [9]. At an excitation wavelength of 485 nm and emission at 530 nm, the dye accumulates in mitochondria with increasing Δψm in monomeric conditions. Cells were washed with serum-free medium and incubated with 10 μM JC-1 at 37°C. Then, cells were resuspended with medium containing 10% serum and fluorescence measured at the two different wavelengths. The data are representative of ten individual experiments.

### Cell viability assay

We measured cell proliferating activity with the water-soluble tetrazolium salt (WST-1) assay (Roche Diagnostics, Mannheim, Germany). The WST-1 assay is a colorimetric method in which the dye intensity is proportional to the number of viable cells. Cells were seeded into 96-well microtiter plates at a concentration of 5 × 10^3^ cells/well. After a 12 h incubation, cells were treated with various conditions for 48 h. After incubation, the cells were washed with PBS and the cell proliferation reagent WST-1 was added, then samples were incubated for 4 h. Sample absorbance was analysed with a bichromatic ELISA reader at 450 nm. All experiments were performed in triplicate with different passages of the C33a and SiHa cells.

### In vitro migration assay

Cell migration was measured using 24 mm diameter chambers with 8 μm pore filters (Transwell, 6-well cell culture). C33a and SiHa cells were collected and resuspended at 7.5 × 10^6^ cells/mL in serum-free medium, and a 0.2 mL cell suspension was added to the upper chambers. Then, the treatment medium (0.5 mL) was added to the lower chambers. The chambers were incubated for 48 h at 37°C in a humidified atmosphere of 5% CO_2_/95% air. Next, the filters were fixed in 95% ethanol and stained with H.E. The upper surfaces of the filters were scraped twice with cotton swabs to remove non-migrated cells. The experiments were repeated in triplicate with different passages of the C33a and SiHa cells, and the migrated cells were counted microscopically (400 ×) in five different fields per filter.

### Detection of apoptotic cells

Annexin V-FITC and PI staining were utilised to detect apoptotic C33a and SiHa cells by flow cytometric analysis. Cells were washed and resuspended with binding buffer before being transferred to a 5 mL tube. The cells were incubated in the dark for 15 minutes after the addition of 5 μL Annexin V and 5 μL PI. Binding buffer was then added to each tube, and the samples were analysed by a Beckman Coulter Epics XL flow cytometer.

### Statistical analysis

Most results are presented as the mean ± standard deviation (SD). Differences between various data sets were tested for significance using a Student’s t-test, and a *p*-value of less than 0.05 was considered significant (***p < 0.001; **p < 0.01; *p < 0.05; ^#^*p* > 0.05).

## Results

### The expression of gC1qR in human cervical tissues

**Figure 1:**
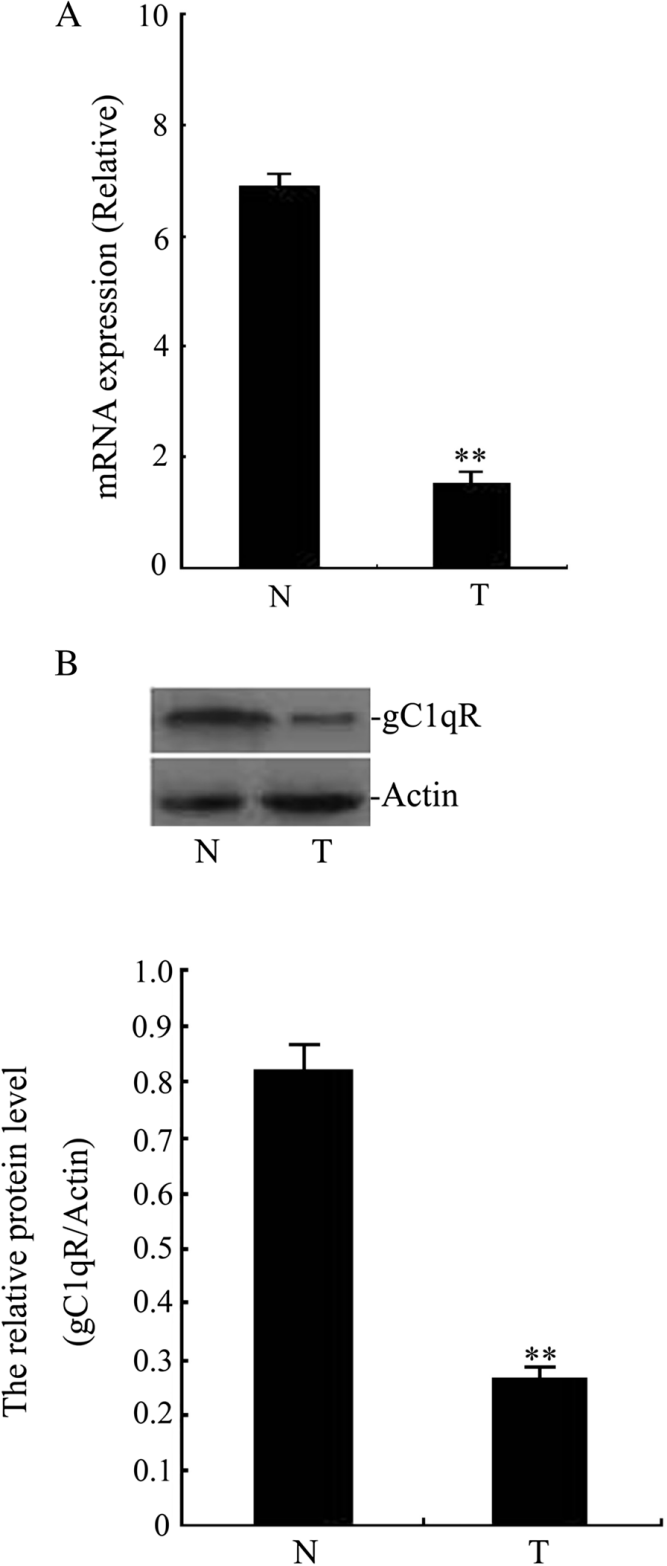
**The levels of gC1qR in cervical tissues.** Relative gC1qR gene and protein levels are shown for human cervical squamous cell carcinoma tissues (T) and surrounding non-neoplastic tissues (N). **A**: The mRNA levels of gC1qR were analysed by real-time PCR. **p < 0.01, significantly different when compared with surrounding non-neoplastic tissues (n = 30). **B**: The levels of gC1qR protein were measured by Western blot analysis. The graph shows the relative gC1qR protein levels, which were quantified and normalised to β-actin. Values are represented as the means ± SD. The gC1qR gene levels were low in human cervical squamous cell carcinoma tissues. **p < 0.01, significantly different when compared with surrounding non-neoplastic tissues (n = 30).

In order to investigate the levels of gC1qR gene and protein expression in human cervical squamous cell carcinomas, 30 cases of human cervical squamous cell carcinomas and surrounding non-neoplastic tissues were analysed by real-time quantitative polymerase chain reaction (real-time PCR) and Western blot (Figure 1). The results showed that the mRNA expression and protein levels of gC1qR were significantly decreased in human cervical squamous cell carcinoma tissues (T) compared with the surrounding non-neoplastic tissues (N). This finding suggested that gC1qR might play a negative role in the survival of human cervical squamous cell carcinoma.

### Comparison of gC1qR expression in human cervical squamous carcinoma cells and human cervical epithelial cells

**Figure 2:**
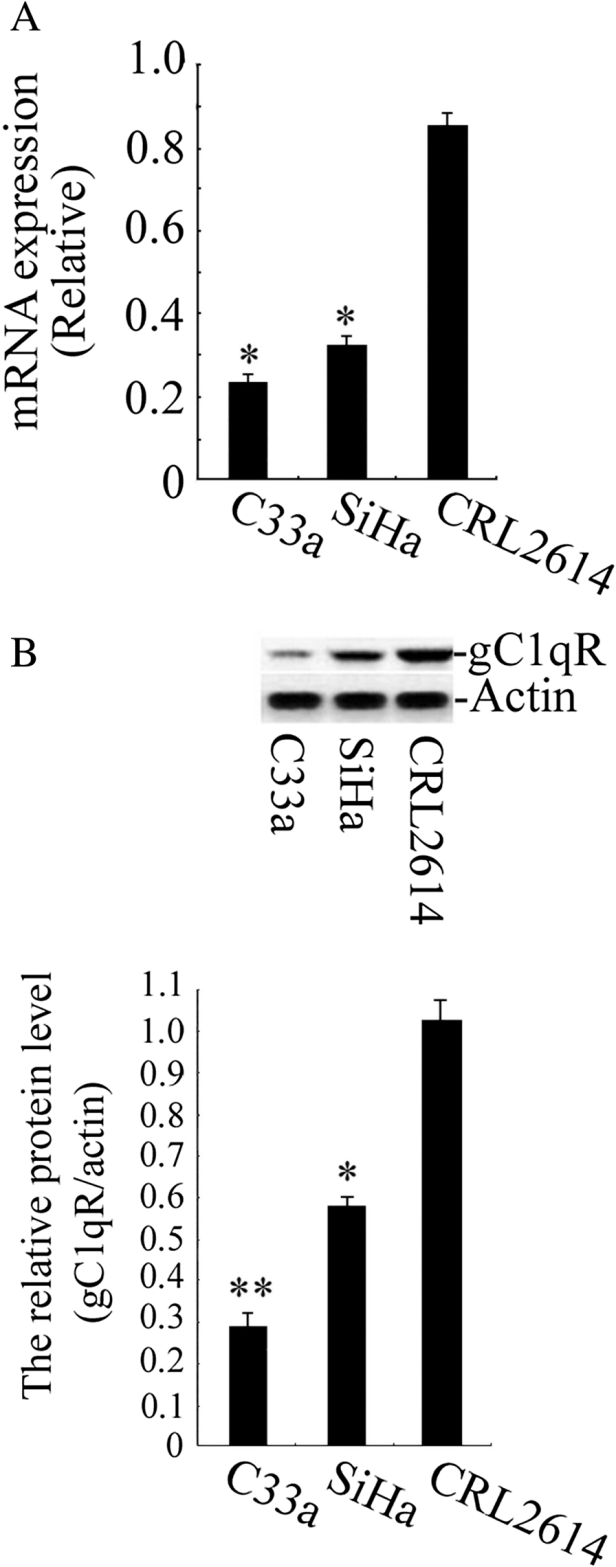
**The levels of gC1qR expression in cell lines. A**: Relative gC1qR gene expression levels are shown in cervical squamous carcinoma cell lines C33a and SiHa and a human cervical epithelial cell line, CRL2614. The expression levels of gC1qR were analysed by real-time PCR. **B**: The levels of gC1qR protein were measured by Western blot. The graph depicts the relative gC1qR protein levels normalised to actin. The results are expressed as the means ± SD of three separate experiments. **p < 0.01 versus CRL2614 cells; * *p* < 0.05 versus CRL2614 cells.

Next, we determined the gC1qR expression levels in two cervical squamous carcinoma cell lines, C33a and SiHa, and a human cervical epithelial cell line, CRL2614 (Figure 2). Real-time PCR and Western blot results showed that the level of gC1qR expression in C33a and SiHa cells was lower than that in CRL2614 cells. Thus, the C33a and SiHa cells were the primary focus of subsequent experiments.

### Functional analysis of the effects of gC1qR treatment on cervical squamous carcinoma cell lines

**Figure 3:**
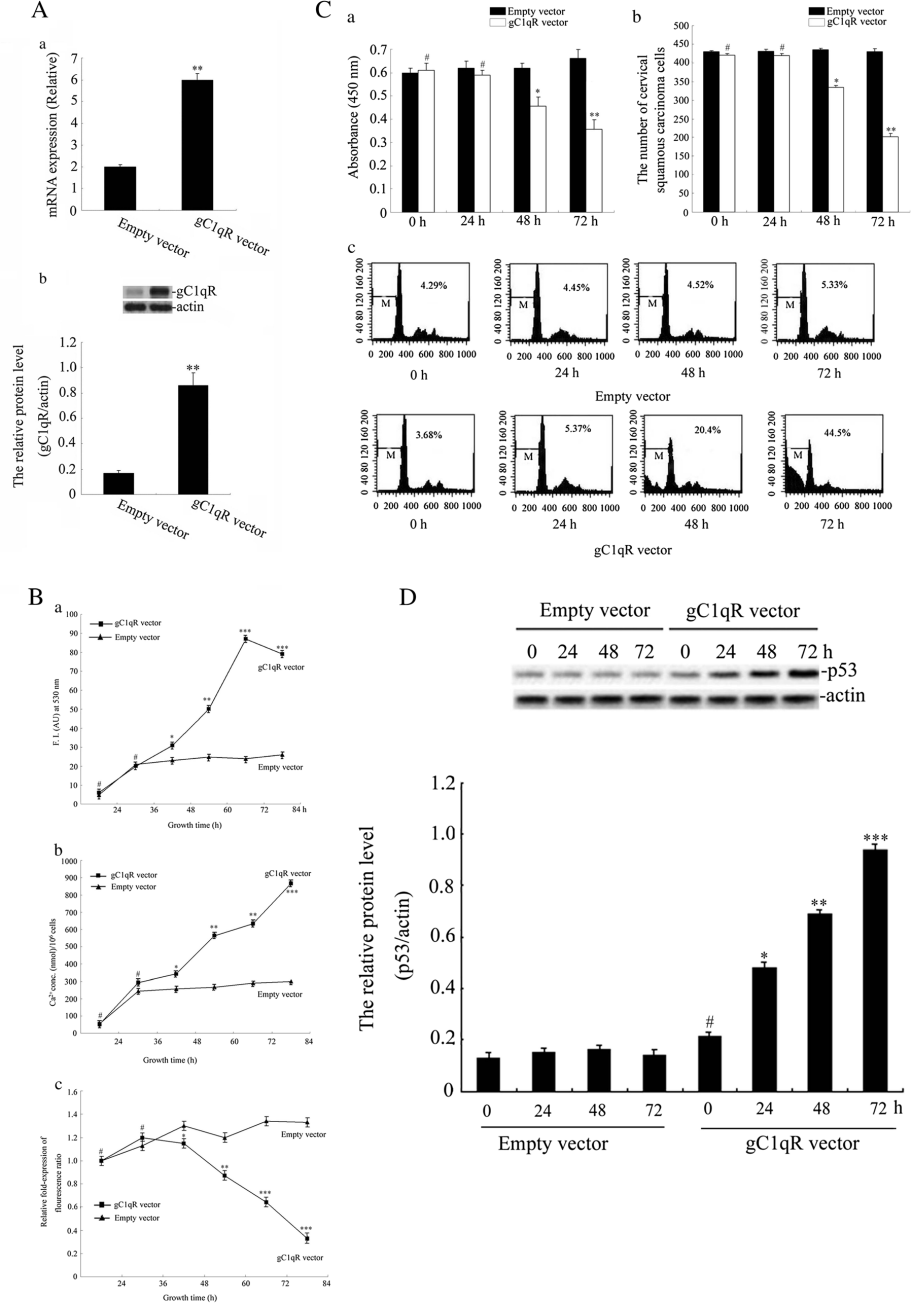
**The biological effects of gC1qR overexpression on C33a and SiHa cells. A, a**: The expression levels of gC1qR mRNA were analysed by real-time PCR. **b**: The levels of gC1qR protein were measured by Western blot analysis. The results are shown the means ± SD of three separate experiments. **p < 0.01 compared with the empty vector group. **B**, **a**: ROS generation was quantified by measuring the fluorescence of H_2_DCFDA for 30 min with fluorescence microscopy. **b**: Intracellular Ca^2+^ levels were monitored using the fluorescence probe fluo-4 AM. **c**: Time-dependent changes in relative Δψm values were measured by the fluorescence of JC-1(590 nm: 527 nm). These data are representative of three separate experiments. *p < 0.05, **p < 0.01, ***p < 0.001, ^#^*p* > 0.05 versus the empty vector group. **C**, **a**: The viability of cells was detected via a WST-1 assay. Sample absorbance was analysed using a bichromatic ELISA reader at 450 nm; **b**: The migration of cells was measured by a Transwell assay. The migrated cells were counted microscopically (400 X) in five different fields per filter. ^#^*p* > 0.05, *p < 0.05, **p < 0.01 versus the empty vector group; **c**: Apoptotic death of C33a and SiHa cells was assessed by flow cytometric analysis. **D**: The level of p53 was measured by Western blot. The results are expressed as the means ± SD of three separate experiments. *p < 0.05, **p < 0.01, ***p < 0.001, ^#^*p* > 0.05 versus the empty vector group.

To explore the function of gC1qR, it was overexpressed in C33a and SiHa cells. Both the mRNA and protein expression of gC1qR were detected in C33a and SiHa cells transfected with either gC1qR vector or empty vector (Figure 3A), the levels of gC1qR were significantly increased after transfection with the gC1qR vector. Using these transfectants, we explored the biological effects of gC1qR overexpression on C33a and SiHa cells. Mitochondrial function was assessed via ROS levels, cytosolic Ca^2+^ levels, and the change in Δψm. After transfection with the gC1qR vector or empty vector for 0, 24, 36, 48, 60, 72, and 84 h, ROS generation was determined by H_2_DCFDA fluorescence. ROS levels in the gC1qR vector group increased in a time-dependent manner, and maximum levels of ROS were detected 72 h after the transfection (Figure 3B-a). As shown Figure 3B-b, cytosolic Ca^2+^ was elevated 84 h after transfection. At this time, the [Ca^2+^]i concentration of the gC1qR vector group was 3.68-fold that of the empty vector group. The elevation of cytosolic Ca^2+^ also occurred in a time-dependent manner. Changes in the relative Δψm values in gC1qR-overexpressing C33a and SiHa cells were also explored over time. We used the JC-1 dye to monitor Δψm, which is determined by the 590 nm: 527 nm emission ratio. The value of Δψm in the gC1qR vector treatment group was approximately 73% less than that of the empty vector group at 84 h. Thus, the Δψm in cervical squamous carcinoma cells overexpressing gC1qR was decreased (Figure 3B-c).

To investigate the effect of gC1qR on human cervical squamous carcinoma cell viability, migration and apoptosis, C33a and SiHa cells were transfected with the gC1qR vector or empty vector. Cell viability was determined by a WST-1 assay. As shown in Figure 3C-a, overexpression of gC1qR decreased cell viability as compared with the empty vector group at 48 h and 72 h. Moreover, the viability of cells in the empty vector group did not change from 0 h to 72 h. The number of migrated cells was greatly decreased in cells overexpressing gC1qR compared with those transfected with the empty vector. The amount of migrated cells in the empty vector group did not change from 0 to 72 h (Figure 3C-b). In addition, to extend our work, the amount of apoptotic death of C33a and SiHa cells was assessed. A typical transfected human cervical squamous carcinoma cell that is undergoing apoptosis is indicated in Figure 3C-c and Additional file 1: Figure S1. The results showed that overexpression of gC1qR increased the number of cervical squamous carcinoma cells in the subG1 region at 72 h. In contrast, the number of subG1 cells in the empty vector-transfected cells showed no change over time.

Overexpression of gC1qR in human cervical squamous carcinoma cells resulted in the production of p53, as shown in Figure 3D, Additional file 2: Figure S2 and Additional file 3: Figure S3. We measured the levels of p53 after transfection with the gC1qR vector or empty vector for 0 h, 24 h, 48 h, and 72 h. These data found that p53 was notably increased in cervical squamous carcinoma cells transfected with gC1qR vector in a time-dependent manner. In contrast, no change in p53 expression was found in cells treated with the empty vector.

### gC1qR affects mitochondrial function in C33a and SiHa cells via a p53-dependent pathway

**Figure 4:**
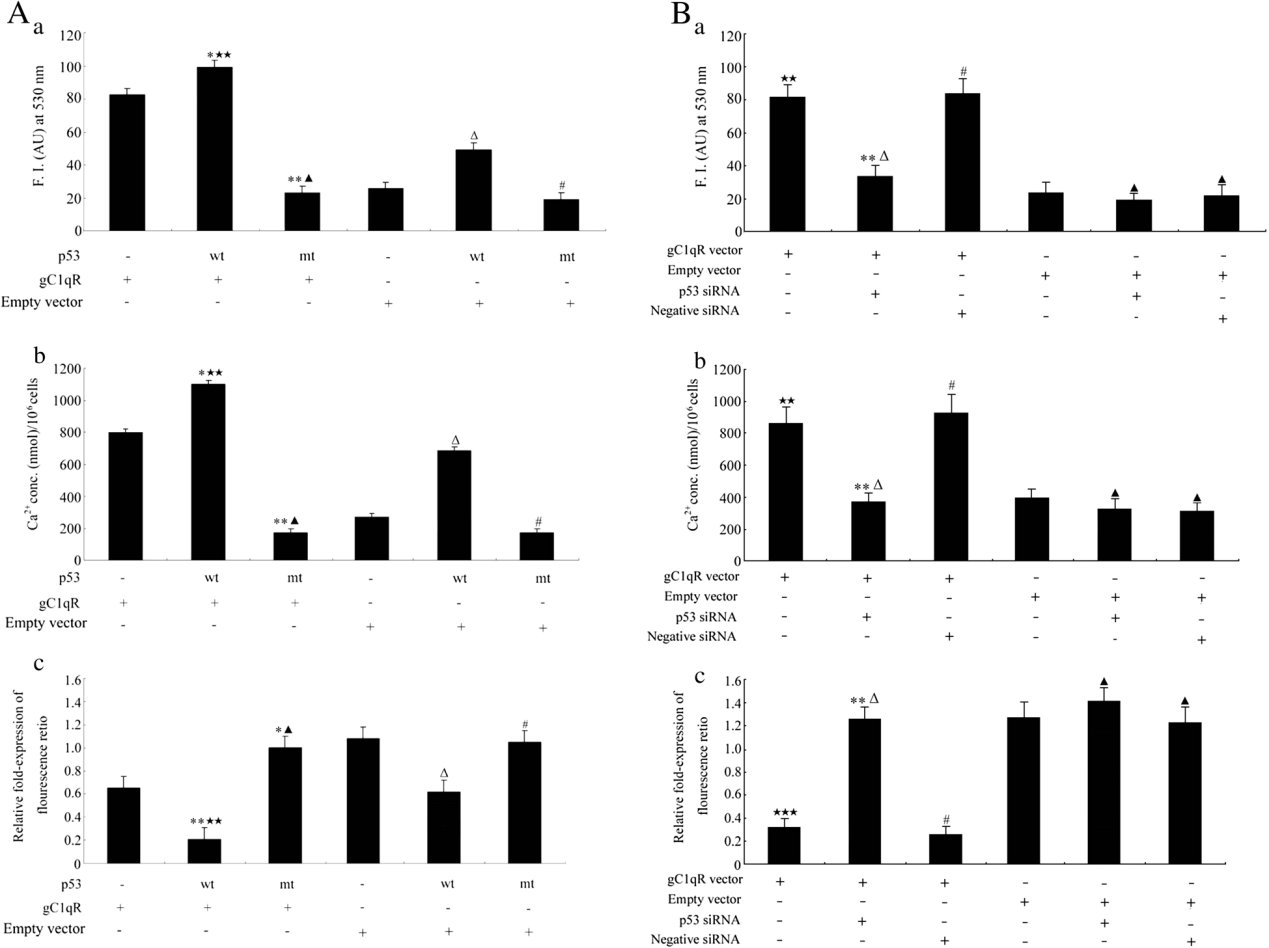
**The analysis of mitochondrial function of C33a and SiHa cells. A, a**: ROS generation was quantified by the fluorescence of H_2_DCFDA for 30 min by fluorescence microscopy; **b**: Intracellular Ca^2+^ levels were monitored using the fluorescence probe fluo-4 AM; **c**: The relative Δψm values were measured by fluorescence of JC-1(590 nm: 527 nm). These data are representative of three separate experiments. *p < 0.05, **p < 0.01 versus p53 (-), gC1qR vector (+) and empty vector (-) group; ^Δ^*p* < 0.05, ^#^*p* > 0.05 versus p53 (-), gC1qR vector (-) and empty vector (+) group; ^★★^*p* < 0.01 versus p53 wt (+), gC1qR vector (-) and empty vector (+) group; ^▲^*p* > 0.05 versus p53 mt (+), gC1qR vector (-) and empty vector (+) group. **B**, **a**: ROS generation was quantified by the fluorescence of H_2_DCFDA for 30 min by fluorescence microscopy; **b**: Intracellular Ca^2±^ levels were monitored using the fluorescence probe fluo-4 AM; **c**: The relative Δψm values were measured by fluorescence of JC-1(590 nm: 527 nm). These data are representative of three separate experiments. **p < 0.01, ^#^*p* > 0.05 versus gC1qR vector (+) group; ^▲^*p* >0.05 versus empty vector (+) group; ^★★^*p* <0.01, ^★★★^*p* < 0.001 versus empty vector (+) group; ^Δ^*p* >0.05 versus empty vector (+) and p53 siRNA (+) group.

Cervical squamous carcinoma cells were transiently transfected with gC1qR vector or empty vector. At 72 h post-transfection, cells were transfected with 100 ng of empty pCB6 vector, which encodes p53 negative (-), 100 ng of pCB+ p53, which encodes wild-type p53 (wt), or 100 ng of pCB6+ p53 173 L, which encodes mutant p53 (mt). Co-expression of wild-type p53 and gC1qR increased the levels of gC1qR-induced ROS production by almost 21.9% at 72 h compared with the empty pCB6 vector + gC1qR vector group. In contrast, co-expression of the trans-dominant negative p53 173 L mutant and gC1qR decreased the level of gC1qR-induced ROS generation by almost 62.2% compared with the empty pCB6 vector + gC1qR vector group. Co-expression of wild-type p53 and empty vector increased ROS production compared with the empty pCB6 vector + empty vector group. The ROS production in the mutant p53 and empty vector group was no change compared with the empty pCB6 vector + empty vector group (p > 0.05). Moreover, the ROS levels was notably increased in cells transfected with the wild-type p53 and gC1qR vector when compared with the wild-type p53 and empty vector group, however, the ROS generation had no apparently different between the p53 173 L mutant + gC1qR vector group and the p53 173 L mutant + empty vector group (p > 0.05) (Figure 4A-a). As shown in Figure 4A-b, the [Ca^2+^]i concentration in cells co-expressing wild-type p53 and gC1qR was 1.38-fold that of the empty pCB6 vector + gC1qR vector group. Furthermore, co-expression of gC1qR and the trans-dominant negative p53 173 L mutant decreased the levels of cytosolic Ca^2+^ by almost 77.5% compared with the empty pCB6 vector + gC1qR vector group. Co-expression of wild-type p53 and empty vector increased [Ca^2+^]i concentration compared with the empty pCB6 vector + empty vector group. The [Ca^2+^]i concentration in the mutant p53 and empty vector group was no change compared with the empty pCB6 vector + empty vector group (p > 0.05). Moreover, the [Ca^2+^]i levels was notably increased in cells transfected with the wild-type p53 and gC1qR vector when compared with the wild-type p53 and empty vector group, however, the [Ca^2+^]i concentration had no apparently different between the p53 173 L mutant + gC1qR vector group and the p53 173 L mutant + empty vector group (p > 0.05). The value of Δψm was approximately 69.2% lower in cells co-expressing wild-type p53 and gC1qR vector compared with the empty pCB6 vector + gC1qR vector group. Furthermore, there was an increase in Δψm in C33a and SiHa cells transfected with the mutant p53 and gC1qR vectors group. Co-expression of wild-type p53 and empty vector decreased the value of Δψm compared with the empty pCB6 vector + empty vector group. The value of Δψm in the mutant p53 and empty vector group was no change compared with the empty pCB6 vector + empty vector group. Moreover, the value of Δψm was notably decreased in cells transfected with the wild-type p53 and gC1qR vector when compared with the wild-type p53 and empty vector group, however, the relative Δψm values had no apparently different between the p53 173 L mutant + gC1qR vector group and the p53 173 L mutant + empty vector group (p > 0.05) (Figure 4A-c).

To determine if accumulated gC1qR affects mitochondrial dysfunction in C33a and SiHa cells via a p53-dependent pathway, cervical squamous carcinoma cells were transiently transfected with gC1qR vector or empty vector. At 72 h post-transfection, cells were transfected with p53 siRNA or negative siRNA (An unrelated gene siRNA as a negative control). Co-expression of gC1qR vector + p53 siRNA decreased the levels of gC1qR-induced ROS production by almost 63.4% at 72 h compared with the gC1qR vector group. However, the ROS production in gC1qR vector + negative siRNA group was no change compared with the gC1qR vector group (p > 0.05), in addition, the ROS generation had no apparently different between the empty vector + p53 siRNA group, empty vector + negative siRNA group and the empty vector group (p > 0.05). In contrast, the ROS levels was notably increased in cells transfected with the gC1qR vector when compared with the empty vector group, however, the ROS production in gC1qR vector + p53 siRNA group was no change compared with the empty vector + p53 siRNA group (Figure 4B-a). As shown in Figure 4B-b, co-expression of gC1qR vector and p53 siRNA decreased the levels of cytosolic Ca^2+^ by almost 67.1% compared with the gC1qR vector group. The [Ca^2+^]i concentration in the gC1qR vector + negative siRNA group was no change compared with the gC1qR vector group (p > 0.05). Moreover, the [Ca^2+^]i concentration had no apparently different between the empty vector + p53 siRNA group, empty vector + negative siRNA group and the empty vector group (p > 0.05). In contrast, the [Ca^2+^]i concentration in cells transfected with gC1qR vector was 2.83-fold that of the empty vector group. Furthermore, the [Ca^2+^]i levels had no apparently different between the gC1qR vector + p53 siRNA group and the empty vector + p53 siRNA group. The value of Δψm was apparently increased in cells co-expressing gC1qR vector and p53 siRNA compared with the gC1qR vector group, but the value of Δψm in the gC1qR vector + negative siRNA group was no change compared with the gC1qR vector group. However, the relative Δψm values had no apparently different between the empty vector + p53 siRNA group, empty vector + negative siRNA group and empty vector group (p > 0.05). The value of Δψm was notably decreased in cells transfected with gC1qR vector when compared with the empty vector group. However, the value of Δψm in the gC1qR vector + p53 siRNA group was no change compared with the empty vector + p53 siRNA group (Figure 4B-c).

### gC1qR overexpression induced biological effects via a p53-dependent pathway in C33a and SiHa cells

**Figure 5:**
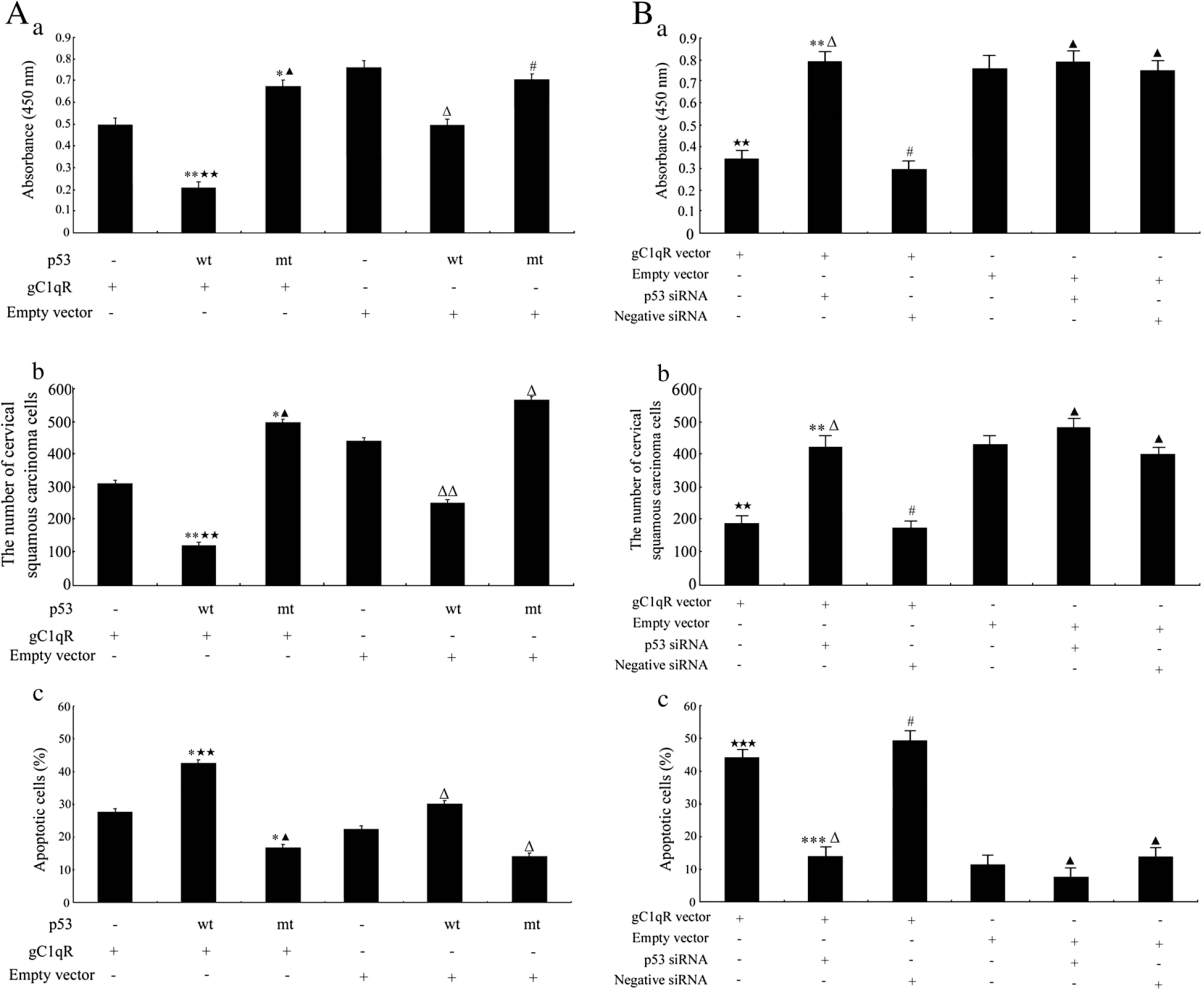
**Viability, migration and apoptosis of C33a and SiHa cells. A, a**: C33a and SiHa cell viability was detected via a WST-1 assay. Sample absorbance was analysed using a bichromatic ELISA reader at 450 nm; **b**: The migration of cells was measured by Transwell assay. The migrated cells were counted microscopically (400 X) in five different fields per filter; **c**: Numbers of apoptotic cells were measured by flow cytometric analysis. These results are shown as the means ± SD from 3 independent experiments. *p < 0.05, **p < 0.01 versus p53 (-), gC1qR vector (+) and empty vector (-) group; ^Δ^*p* < 0.05, ^δδ^*p* < 0.01, ^#^*p* > 0.05 versus p53 (-), gC1qR vector (-) and empty vector (+) group; ^★★^*p* < 0.01 versus p53 wt (+), gC1qR vector (-) and empty vector (+) group; ^▲^*p* > 0.05 versus p53 mt (+), gC1qR vector (-) and empty vector (+) group. **B**, **a**: C33a and SiHa cell viability was detected via a WST-1 assay; **b**: The migration of cells was measured by Transwell assay; **c**: Numbers of apoptotic cells were measured by flow cytometric analysis. These results are shown as the means ± SD from 3 independent experiments. ***p < 0.001, **p < 0.01, ^#^*p* > 0.05 versus gC1qR vector (+) group; ^▲^*p* > 0.05 versus empty vector (+) group; ^★★^*p* < 0.01, ^★★★^*p* < 0.001 versus empty vector (+) group; ^Δ^*p* > 0.05 versus empty vector (+) and p53 siRNA (+) group.

To investigate the effect of p53 on viability, migration and apoptosis in gC1qR-overexpressing human cervical squamous carcinoma cells, C33a and SiHa cells were transfected with gC1qR vector or empty vector, at 72 h post-transfection, cells were transfected with 100 ng of empty pCB6 vector, which encodes p53 negative (-), 100 ng of pCB+ p53, which encodes wild-type p53 (wt), or 100 ng of pCB6+ p53 173 L, which encodes mutant p53 (mt). As shown in Figure 5A-a, co-expression of wild-type p53 and gC1qR decreased cell viability compared with transfection with the empty pCB6 vector + gC1qR vector group, the viability of cells in the mutant p53 and gC1qR vector group was increased compared with the empty pCB6 vector + gC1qR vector group. Co-expression of wild-type p53 and empty vector decreased the viability of cells compared with the empty pCB6 vector + empty vector group. The viability of cells in the mutant p53 and empty vector group was no change compared with the empty pCB6 vector + empty vector group. Moreover, cell viability was notably decreased in cells transfected with the wild-type p53 and gC1qR vector when compared with the wild-type p53 and empty vector group, in contrast, the viability of cells had no apparently different between the p53 173 L mutant + gC1qR vector group and the p53 173 L mutant + empty vector group (p > 0.05). The number of migrated cells was significantly lower in the cells transfected with wild-type p53 and gC1qR vectors compared with the empty pCB6 vector + gC1qR vector group (*p* < 0.01). The numbers of migrated cells were different between mutant p53 + gC1qR vector group and empty pCB6 vector + gC1qR vector group. Co-expression of wild-type p53 and empty vector decreased the number of migrated cells compared with the empty pCB6 vector + empty vector group. The number of migrated cells in the mutant p53 and empty vector group was different compared with the empty pCB6 vector + empty vector group. Moreover, migrated cells were notably decreased in cells transfected with the wild-type p53 and gC1qR vector when compared with the wild-type p53 and empty vector group, however, the number of migrated cells had no apparently different between the p53 173 L mutant + gC1qR vector group and the p53 173 L mutant + empty vector group (p > 0.05) (Figure 5A-b). As shown in Figure 5A-c, expression of the wild-type p53 and gC1qR vector increased the apoptosis of C33a and SiHa cells. The apoptosis of cervical squamous carcinoma cells in the group treated with the mutant p53 + gC1qR vector was lower than that in the empty pCB6 vector + gC1qR vector group (*p* < 0.05). Co-expression of wild-type p53 and empty vector increased apoptosis cells compared with the empty pCB6 vector + empty vector group. Apoptosis cells in the mutant p53 and empty vector group was different compared with the empty pCB6 vector + empty vector group. Moreover, apoptosis cells were notably increased in cells transfected with the wild-type p53 and gC1qR vector when compared with the wild-type p53 and empty vector group, however, there was no different in cell apoptosis between the p53 173 L mutant + gC1qR vector group and the p53 173 L mutant + empty vector group (p > 0.05).

To explore if accumulated gC1qR affects viability, migration and apoptosis in C33a and SiHa cells via a p53-dependent pathway, human cervical squamous carcinoma cells were transfected with gC1qR vector or empty vector, at 72 h post-transfection, cells were transfected with p53 siRNA or negative siRNA. As shown in Figure 5B-a, co-expression of gC1qR vector and p53 siRNA increased cell viability compared with transfection with the gC1qR vector group, the viability of cells had no apparently different between the gC1qR vector group and the gC1qR vector + negative siRNA group (p > 0.05). Moreover, the viability of cells in the empty vector + p53 siRNA group, empty vector + negative siRNA group was no change compared with the empty vector group. The viability of cells in the gC1qR vector group was decreased compared with the empty vector group. However, there was no difference between the gC1qR vector + p53 siRNA group and the empty vector + p53 siRNA group. The number of migrated cells was significantly increased in the cells transfected with gC1qR vectors and p53 siRNA compared with the gC1qR vector group (*p* < 0.01). The numbers of migrated cells were no difference between the gC1qR vector + negative siRNA group and the gC1qR vector group. Moreover, the number of migrated cells had no apparently different between the empty vector + p53 siRNA group, the empty vector + negative siRNA group and the empty vector group (p > 0.05). Migrated cells were notably decreased in cells transfected with the gC1qR vector when compared with the empty vector group. However, the number of migrated cells in the gC1qR vector and p53 siRNA group had no change compared with the empty vector + p53 siRNA group (Figure 5B-b). As shown in Figure 5B-c, expression of the gC1qR vector and p53 siRNA decreased the apoptosis of C33a and SiHa cells. The apoptosis of cervical squamous carcinoma cells in the group treated with the gC1qR vector + negative siRNA had no difference compared with the gC1qR vector group (*p* > 0.05). Moreover, apoptosis cells in the empty vector + p53 siRNA group, empty vector + negative siRNA group were no different compared with the empty vector group. Apoptosis cells were notably increased in cells transfected with the gC1qR vector when compared with the empty vector group, however, there was no different in cell apoptosis between the gC1qR vector + p53 siRNA group and the empty vector + p53 siRNA group (p > 0.05). Taken together, these results strongly suggest that p53 activity is required for gC1qR-induced apoptotic cervical cancer cell death.

## Discussion

Apoptosis is a programmed cell death process, which is a critical protective mechanism against carcinogenesis [10]. Therefore, induction of apoptotic cell death is a promising strategy for the prevention of cancer. Our goals in these experiments were to demonstrate that gC1qR strongly induced ROS production in mitochondria, and that this oxidative stress induced apoptosis through a p53-dependent pathway in cervical squamous carcinoma cells. The relationship between mitochondrial dysfunction, p53 status and gC1qR expression in the regulation of apoptosis has not been examined. We have shown previously that gC1qR is capable of inducing apoptosis in human cervical squamous carcinoma cells [11]. These findings constitute the first evidence for the mitochondria as a target of gC1qR-induced apoptosis in a p53-dependent fashion.

gC1qR is a multi-compartmental and multibinding, and thus multifunctional, cellular protein that is expressed in a wide range of tissues and cell types [12]. In the present study, our data demonstrated that gC1qR had low expression in human cervical squamous carcinoma tissues. The list of biologic responses mediated by gC1qR is extensive, including roles in inflammation, infection and immune regulation [13,14]. When constitutively expressed in a normal murine fibroblast cell line, gC1qR induces growth perturbation, morphological abnormalities and apoptosis [15]. gC1qR has been extensively studied in a previous study, primarily as an inducer of apoptosis [16]. In the present study, we found that overexpression of gC1qR in cervical squamous carcinoma cells results in increased rates of apoptosis.

Recent cohort studies have shown that gC1qR is a conserved eukaryotic multifunctional protein that primarily localised in the mitochondrial matrix and on the cell surface [17]. Human gC1qR is expressed as a proprotein of 282 amino acids (aa) whose first 73 amino acids, containing a mitochondrial localization signal, are required for localizing the protein to the mitochondria and are subsequently cleaved to generate mature gC1qR. The mature form of gC1qR has been tied to apoptosis and autophagy via inducing mitochondrial dysfunction [18]. Increasing evidence suggests that mitochondrial dysfunction is linked to apoptosis initiated by cytotoxic factors such as ROS, which are generated in excess in defective mitochondria. These findings have focused attention on the role of the mitochondria in apoptosis. While it is not yet clear how mitochondria regulate apoptosis, it has been suggested that a megachannel of high conductance results in the loss of mitochondrial membrane potential and mitochondrial function [19,20]. Our study demonstrated that gC1qR-treated C33a and SiHa cells overexpressing gC1qR resulted in the generation of ROS, which correlated with intracellular Ca^2+^ accumulation. Thus, the continuation of ROS generation in gC1qR-overexpressing cells was associated with intracellular Ca^2+^ accumulation, which may lead to mitochondrial dysfunction. It was expected that interference with electron transport by ROS and increased intracellular Ca^2+^ would influence mitochondrial membrane potential. Indeed, loss in Δψm occurred in C33a and SiHa cells overexpressing gC1qR. These data support our theory that mitochondrial Ca^2+^ overload occurs in gC1qR-overexpressing cells, followed by apoptosis due to Ca^2+^ accumulation in the mitochondria, which suggests a role for gC1qR in mitochondrial-dependent apoptosis.

In this report, we also demonstrated that p53 activity is required downstream of gC1qR in this apoptotic pathway. These findings suggest that the association between these two proteins might have an important role and opens a new field of investigation into the function of p53 in the biology of gC1qR-induced apoptosis of human cervical squamous carcinoma cells. There is evidence to suggest that gC1qR-induced apoptosis occurs via both a p53-dependent and a p53-independent pathway [16]. We showed that in C33a and SiHa cells, overexpression of gC1qR induces apoptosis; moreover, co-expression of wild-type p53 and gC1qR resulted in decreased viability and less gC1qR-induced migration. However, gC1qR inhibition of viability and migration can be abrogated by co-expression of a transdominant negative mutant of p53 or silence the gene of p53. The ratio of the pro-apoptotic Bax and the anti-apoptotic Bcl-2 ultimately determines whether a cell survives or dies through the mitochondrial death pathway [21]. Our data suggest that gC1qR-induced apoptosis is regulated principally by p53, and, accordingly, the ratio of Bax/Bcl-2 was decreased when cells were transfected with gC1qR and mutant p53 (data not shown). These findings strongly suggest that gC1qR induces apoptosis through a p53-dependent pathway in cervical squamous carcinoma cells.

## Conclusion

We demonstrate that gC1qR plays an important role in cervical cancer cell apoptosis, that increased levels of gC1qR are important in this apoptosis and that gC1qR induces apoptosis through the mitochondrial pathway and p53-dependent pathway in human cervical squamous carcinoma cells.

## Abbreviations

gC1qR: Globular heads of C1q receptor; ROS: Reactive oxygen species; Δψm: Mitochondrial membrane potential; siRNA: Small interference RNA; SOCS: Suppressor of cytokine signaling; PI: Propidium iodide; cDNA: Complementary DNA; WST-1: Water-soluble tetrazolium salt; SD: Standard deviation; real-time PCR: Real-time quantitative polymerase chain reaction; wt: Wild-type; mt: Mutant; H_2_DCFDA: 2', 7'-dichlorodihydrofluorescein diacetate.

## Competing interests

The authors declare that they have no competing interests.

## Authors’ contributions

LJG conceived of the study and drafted the manuscript. ZLC participated in its design and helped to draft the manuscript. PQG carried out the molecular biological studies and performed the statistical analysis. KSL collected the patient information. YJS helped to revise the manuscript and performed the statistical analysis. All authors read and approved the final manuscript.

## Supplementary Material

Additional file 1 **Figure S1.** gC1qR induced cell apoptosis. Cervical squamous carcinoma cell line, C33a, SiHa and human cervical epithelial cell line, CRL2614 were treated with gC1qR vector or empty vector, respectively. At 48 h post-transfection, cells were subjected to flow cytometric analysis to detect apoptotic death. Apoptotic cells were quantitated by the percentage of cells with subG1 DNA content. **p < 0.01, ^#^*p* > 0.05 versus the corresponding empty vector. Click here for file

Additional file 2 **Figure S2.** gC1qR induced the expression of p53. C33a and SiHa cells were transfected with the empty vector or gC1qR vector. At 0 h, 24 h, 48 h, and 72 h post-transfection, the mRNA levels of p53 were analysed by real-time PCR. The results are expressed as the means ± SD of three separate experiments. *p < 0.05, **p < 0.01, ***p < 0.001 significantly different when compared with empty vector group. Click here for file

Additional file 3 **Figure S3.** The levels of p53 expression. A: Relative p53 gene expression levels are shown in cervical squamous carcinoma cell line, C33a, SiHa and human cervical epithelial cell line, CRL2614. The different expression level of p53 were analysed by real-time PCR as described. ^**^p < 0.01 versus CRL2614 cells; B: The expression of the p53 protein was measured by Western blot analysis. The graph depicts the relative p53 protein levels normalised to actin. The results are expressed as the mean ± SD of three separate experiments. ^**^p < 0.01 versus CRL2614 cells. Click here for file

## References

[B1] Clare J Edwards D Bagnall H Pearmain P Lawrence G The use of cervical screening history data to interpret cervical cancer incidence trends J Public Health (Oxf) 2008 30 171 177 10.1093/pubmed/fdn008 18296455

[B2] Gao LJ Guo SY Cai YQ Gu PQ Su YJ Gong H Liu Y Chen C Cooperation of decay-accelerating factor and membrane cofactor protein in regulating survival of human cervical cancer cells BMC Cancer 2009 9 384 388 10.1186/1471-2407-9-384 19878546 PMC2774863

[B3] Patel S Chiplunkar S Host immune responses to cervical cancer Curr Opin Obstet Gynecol 2009 21 54 59 10.1097/GCO.0b013e32831a9890 19125004

[B4] Dedio J Jahnen-Dechent W Bachmann M Muller-Esterl W The multiligand-binding protein gC1qR, putative C1q receptor, is a mitochondrial protein J Immunol 1998 160 3534 3542 9531316

[B5] Rubinstein DB Stortchevoi A Boosalis M Ashfag R Ghebrehiwet B Peerschke EI Calvo F Guillaume T Receptor for the globular heads of C1q (gC1q-R; p33; Hyaluronan-binding protein is preferentially expressed by adenocarcinoma cells Internl Cancer Res 2004 110 741 750 10.1002/ijc.20105 15146564

[B6] Yao ZQ Waggoner SN Cruise MW Hall C Xie X Oldach DW Hahn YS SOCS1 and SOCS3 are targeted by hepatitis C virus core/gC1qR ligation to inhibit T-cell function J Virol 2005 79 15417 15429 10.1128/JVI.79.24.15417-15429.2005 16306613 PMC1315996

[B7] Peerschke EIB Minta JO Zhou SZ Bini A Gotlieb A Colman RW Ghebrehiwet B Expression of gC1qR/p33 and its major ligands in human atherosclerotic lesions Mol Immunol 2004 41 759 766 10.1016/j.molimm.2004.04.020 15234555

[B8] Meenakshi J Anupama SK Datta K Constitutive expression of hyaluronan binding protein 1 (HABP1/p32/gC1qR) in normal fibroblast cells perturbs its growth characteristics and induces apoptosis Biochem Biophys Res Commun 2003 300 686 693 10.1016/S0006-291X(02)02788-2 12507504

[B9] Dey R Moraes CT Lack of oxidative phosphorylation and low mitochondrial membrane potential decrease susceptibility to apoptosis and do not modulate the protective effect of Bcl-x(L) in osteosarcoma cells J Biol Chem 2000 275 7087 7094 10.1074/jbc.275.10.7087 10702275

[B10] Lowe SW Lin AW Apoptosis in cancer Carcinogenesis 2000 21 485 495 10.1093/carcin/21.3.485 10688869

[B11] Gao LJ Gu PQ Fan WM Liu Z Qiu F Peng YZ Guo XR The role of gC1qR in regulating survival of human papillomavirus 16 oncogene-transfected cervical cancer cells In J Oncol 2011 39 1265 1272 10.3892/ijo.2011.1108 21725590

[B12] Peerschke EI Murphy TK Ghebrehiwet B Activation-dependent surface expression of gC1qR/p33 on human blood platelets Thromb Haemost 2003 89 331 339 12574814

[B13] Peerschke EI Ghebrehiwet B The contribution of gC1qR/p33 in infection and inflammation Immunobiology 2007 212 333 342 10.1016/j.imbio.2006.11.011 17544818 PMC2001281

[B14] Eisen-Vandervelde AL Waggoner SN Yao ZQ Cale EM Hahn CS Hahn YS Hepatitis C virus core selectively suppresses interleukin-12 synthesis in human macrophages by interfering with AP-1 activation J Biol Chem 2004 279 43479 43486 10.1074/jbc.M407640200 15292184

[B15] Meenakshi J Anupama Goswami SK Datta K Constitutive expression of hyaluronan binding protein 1 (HABP1/p32/gC1qR) in normal fibroblast cells perturbs its growth characteristics and induced apoptosis Biochem Biophys Res Commun 2003 300 686 693 10.1016/S0006-291X(02)02788-2 12507504

[B16] Kamal A Datta K Upregulation of hyaluronan binding protein 1 (HABP1/p32/gC1qR) is associated with Cisplatin induced apoptosis Apoptosis 2006 11 861 874 10.1007/s10495-006-5396-4 16544101

[B17] Vegh Z Goyarts EC Rozengarten K Mazumder A Ghebrehiwet B Maturation-dependent expression of C1q binding protein on the cell surface of human monocyte-derived dendritic cells Int Immunopharmacol 2003 3 39 51 10.1016/S1567-5769(02)00211-4 12538033

[B18] Muta T Kang D Kitajima S Fujiwara T Hamasaki N p32 protein, a splicing factor 2-associated protein, is localized in mitochondrial matrix and is functionally important in maintaining oxidative phosphorylation J Biol Chem 1997 272 24363 24370 10.1074/jbc.272.39.24363 9305894

[B19] Xu L Xiao N Liu F Ren H Gu J Inhibition of RIG-I and MDA5-dependent antiviral response by gC1qR at mitochondria Proc Natl Acad Sci USA 2009 106 1530 1535 10.1073/pnas.0811029106 19164550 PMC2635802

[B20] Chowdhury AR Ghosh I Datta K Excessive reactive oxygen species induces apoptosis in fibroblasts: role of mitochondrially accumulated hyaluronic acid binding protein 1 (HABP1/p32/gC1qR) Exp Cell Res 2008 314 651 667 10.1016/j.yexcr.2007.10.033 18166172

[B21] Nho KJ Chun JM Kim HK Ethanol extract of Dianthus chinensis L. induces apoptosis in human hepatocellular carcinoma HepG2 cells in vitro Evid Based Complement Alternat Med 2012 201 2 573527 22645629 10.1155/2012/573527 PMC3356935

