# Supplementary figures and images for: The globular heads of the C1q receptor regulate apoptosis in human cervical squamous carcinoma cells via a p53-dependent pathway
Source: J Transl Med. 2012 Dec 26;10:255. doi: 10.1186/1479-5876-10-255 (PMC3567992; doi:10.1186/1479-5876-10-255)

Supplementary Fig. 1

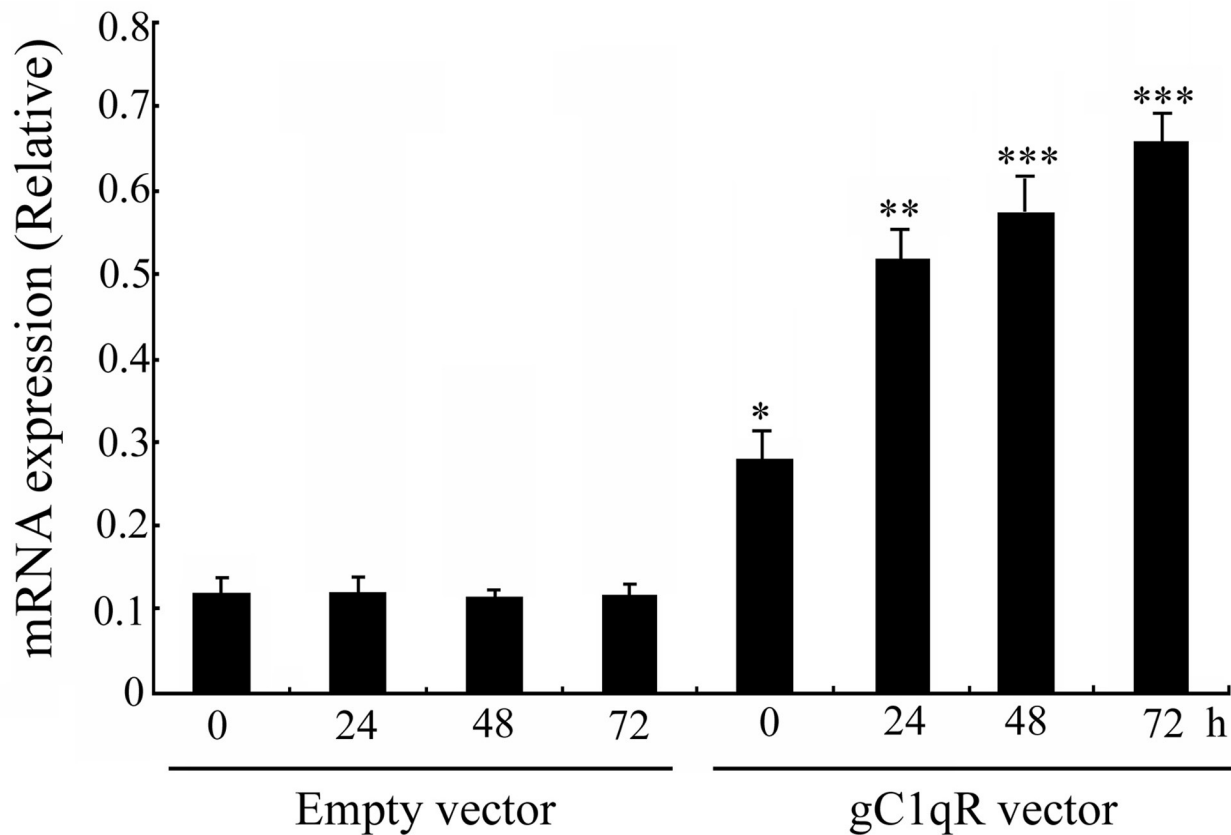

Supplement: Additional file 1 — Figure S1. gC1qR induced cell apoptosis. Cervical squamous carcinoma cell line, C33a, SiHa and human cervical epithelial cell line, CRL2614 were treated with gC1qR vector or empty vector, respectively. At 48 h post-transfection, cells were subjected to flow cytometric analysis to detect apoptotic death. Apoptotic cells were quantitated by the percentage of cells with subG1 DNA content. **p < 0.01, #p > 0.05 versus the corresponding empty vector. [file 1479-5876-10-255-S1.pdf]

Supplementary Fig. 3

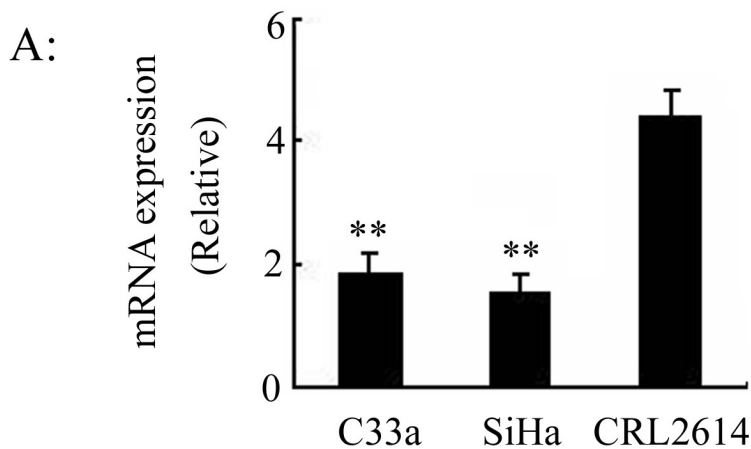

B:

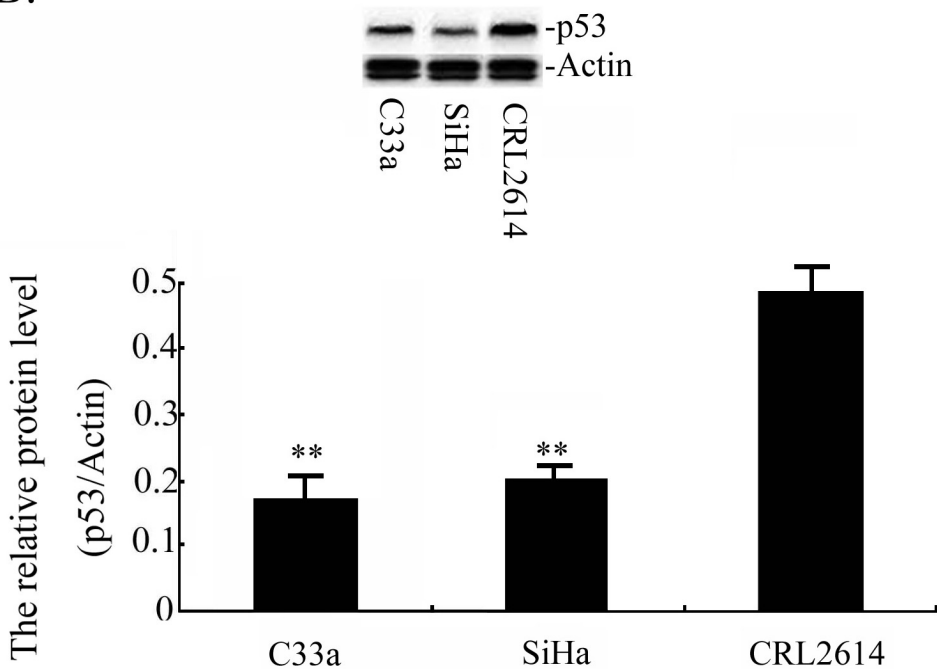

Supplement: Additional file 3 — Figure S3. The levels of p53 expression. A: Relative p53 gene expression levels are shown in cervical squamous carcinoma cell line, C33a, SiHa and human cervical epithelial cell line, CRL2614. The different expression level of p53 were analysed by real-time PCR as described. **p < 0.01 versus CRL2614 cells; B: The expression of the p53 protein was measured by Western blot analysis. The graph depicts the relative p53 protein levels normalised to actin. The results are expressed as the mean ± SD of three separate experiments. **p < 0.01 versus CRL2614 cells. [file 1479-5876-10-255-S3.pdf]
