# Supplementary material for: The globular heads of the C1q receptor regulate apoptosis in human cervical squamous carcinoma cells via a p53-dependent pathway
Source: J Transl Med. 2012 Dec 26;10:255. doi: 10.1186/1479-5876-10-255 (PMC3567992; doi:10.1186/1479-5876-10-255)

Supplementary Fig. 2

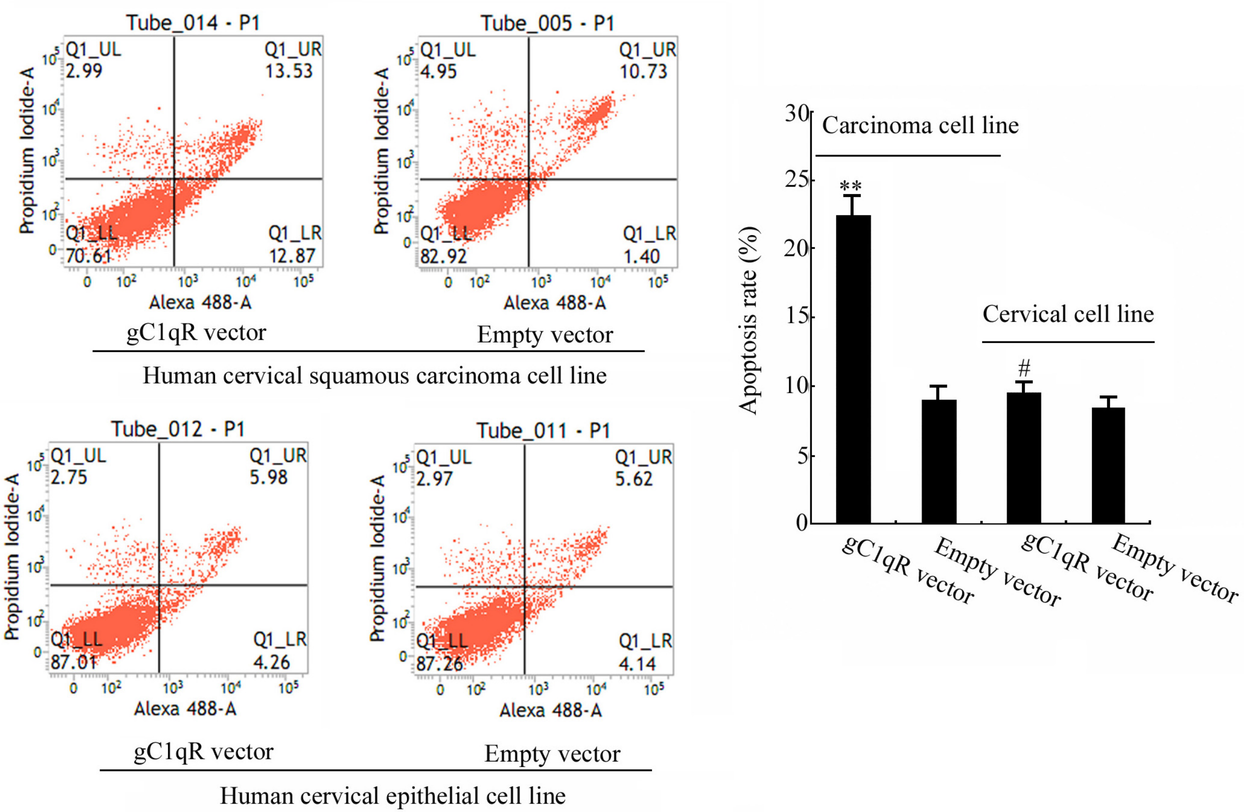

Supplement: Additional file 2 — Figure S2. gC1qR induced the expression of p53. C33a and SiHa cells were transfected with the empty vector or gC1qR vector. At 0 h, 24 h, 48 h, and 72 h post-transfection, the mRNA levels of p53 were analysed by real-time PCR. The results are expressed as the means ± SD of three separate experiments. *p < 0.05, **p < 0.01, ***p < 0.001 significantly different when compared with empty vector group. [file 1479-5876-10-255-S2.pdf]
